# S1P/S1PR3 signalling axis protects against obesity-induced metabolic dysfunction

**DOI:** 10.1080/21623945.2021.2021700

**Published:** 2022-01-30

**Authors:** Sagarika Chakrabarty, Quyen Bui, Leylla Badeanlou, Kelly Hester, Jerold Chun, Wolfram Ruf, Theodore P Ciaraldi, Fahumiya Samad

**Affiliations:** a Department of Cell Biology, San Diego Biomedical Research Institute, San Diego, CA, USA; b Degenerative Diseases Program, Sanford Burnham Prebys Medical Discovery Institute, La Jolla, CA, USA; c Department of Immunology and Microbiology, Scripps Research, La Jolla, Ca and Center for Thrombosis and Hemostasis, University Medical Center, Mainz, Germany; d Department of Medicine, Veterans Affairs San Diego Healthcare System, San Diego, CA, USA; e Department of Medicine, University of California San Diego, La Jolla, CA, USA

**Keywords:** Sphingosine 1 phosphate, S1PR3, obesity, T2D, adipose inflammation, adipogenesis, hepatic steatosis, hepatic inflammation

## Abstract

Sphingosine-1-phosphate (S1P) is a bioactive sphingolipid that interacts via 5 G-protein coupled receptors, S1PR1-5, to regulate signalling pathways critical to biological processes including cell growth, immune cell trafficking, and inflammation.We demonstrate that in Type 2 diabetic (T2D) subjects, plasma S1P levels significantly increased in response to the anti-diabetic drug, rosiglitazone, and, S1P levels correlated positively with measures of improved glucose homeostasis. In HFD-induced obese C57BL/6 J mice S1PR3 gene expression was increased in adipose tissues (AT) and liver compared with low fat diet (LFD)-fed counterparts. On a HFD, weight gain was similar in both S1PR3-/- mice and WT littermates; however, HFD-fed S1PR3-/- mice exhibited a phenotype of partial lipodystrophy, exacerbated insulin resistance and glucose intolerance. This worsened metabolic phenotype of HFD-fed S1PR3-/- mice was mechanistically linked with increased adipose inflammation, adipose macrophage and T-cell accumulation, hepatic inflammation and hepatic steatosis. In 3T3-L1 preadipocytes S1P increased adipogenesis and S1P-S1PR3 signalling regulated the expression of PPARγ, suggesting a novel role for this signalling pathway in the adipogenic program. These results reveal an anti-diabetic role for S1P, and, that S1P-S1PR3 signalling in the adipose and liver defends against excessive inflammation and steatosis to maintain metabolic homeostasis at key regulatory pathways.

## 1. Introduction

Obesity, which has reached pandemic proportions worldwide, contributes to the pathogenesis of insulin resistance (IR), a condition that preceeds Type 2 diabetes (T2D). In the United States, nearly 9.3% of the population is affected by T2D [1], and by 2050 its prevalence is expected to increase to 25% [2]. However, treatment options remain limited, in part due to the challenge in identifying suitable drug targets among the complex series of signalling pathways and cellular mediators that control these disease processes. Adipose tissue (AT) lies at the heart of obesity-mediated health complications. Inflammation of AT, driven by hypertrophic adipocytes and amplified by infiltrating AT macrophages (ATM) and other immune cells causes adipose dysfunction in part by diminishing pre-adipocyte proliferation and differentiation and inducing local adipose IR [3–5]. This dysfunctional AT promotes ectopic lipid deposition in non-AT including the liver, skeletal muscle and pancreas leading to lipotoxicity and metabolic dysfunction in these tissues and to systemic IR and T2D [6,7]. Identifying clinically relevant targets that maintain the production of metabolically healthy adipocytes and limits chronic adipose inflammation is key for developing new treatments for these metabolic disorders.

Obesity and IR are often associated with dysregulation of lipid metabolism and lipotoxicity which interefere with crucial cell signalling pathways of glucose and lipid homeostasis. Lipotoxicity and IR leads to T2D, hepatic steatosis and cardiovascular disease, and, dysregulated sphingolipid metabolism has been linked to the development of these disorders [8–14]. Among the various sphingolipids, ceramide has received the most attention, and numerous studies in humans and rodents indicate the contribution of tissue and plasma ceramides in obesity and its associated comorbidities of IR, hepatic steatosis and T2D [11,12,14–18]. While these studies used pharmacological and genetic inhibition of pathways of ceramide generation and/or degradation, they often also result in changes in other interrelated sphingolipids, such as, sphingosine-1 phosphate (S1P), which has received less attention. S1P, a downstream metabolite of ceramide is a pro-survival bioactive sphingolipid and a critical regulator of signalling pathways central to a number of biological processes including cell growth, immune cell trafficking, and inflammation [19,20]. S1P is generated by phosphorylation of the downstream metabolite of ceramide, sphingosine, by two sphingosine kinases, SK1 and SK2 and has both intracellular and intercellular signalling effects [21,22]. The majority of secreted S1P is derived from SK1 and mediates its functions primarily through five cell-surface G protein-coupled receptors, S1P receptor 1–5 (S1PR1–5), a class of receptors that is highly amenable to pharmacologic manipulation, and, whose distribution varies in tissue specific and temporal fashion with multiple and opposing downstream effects [19,22–24]. The impact of S1P in any given tissue or cell therefore will ultimately be governed by the expression levels of its receptors. However, the role of S1P and its receptors in obesity and associated disease sequelae is not well defined.

Here, we identify a protective role for plasma S1P in IR and T2D. We show that plasma S1P levels were significantly increased in T2D subjects in response to the anti-diabetic drug, rosiglitazone, and, plasma S1P correlates with measures of improved insulin sensitivity and glucose homeostasis. Insulin resistance and glucose intolerance was exacerbated in HFD-fed S1PR3^−/−^ mice, suggesting a protective role for S1P-S1PR3 signalling axis in regulation of metabolic homeostasis. Mechanistically, S1P-S1PR3 signalling has pro-adipogenic and anti-inflammatory functions in adipose tissue, and, in the liver anti-inflammatory and anti-steatosis effects.

## 2. Materials and methods

### 2.1. Animals

Experiments were approved by the Institutional Animal Care and Use Committees (Scripps Research, La Jolla, CA and San Diego Biomedical Research Institute, San Diego, CA). C57BL/6 J mice were from the Jackson Laboratory (Bar Harbour, ME) and the C57BL/6 J S1PR3-deficient (S1PR3^−/−^) mice and WT littermates were bred from pairs supplied by Dr. Jerold Chun (The Scripps Research Institute, La Jolla, CA). In appropriate experiments, male mice were fed a palmitate-rich HFD (60% kcal from fat; Research Diets; D12492) or LFD (10% kcal from fat; Research Diets; D12450B) for 16 wks beginning at 6–8 weeks of age and body weights were monitored weekly. Twenty-four hour food intake was measured as the difference in weight between the food supplied to the cage and what remains at the end of 24 hours.

### 2.2. Human subjects

Human subject protocols were approved by the Committee on Human Investigation at the University of California, San Diego. Subjects were recruited from diabetes clinics and classified as diabetic by their response to a 75-g oral glucose tolerance test according to the American Diabetes Association criteria. In this intervention study, subjects were excluded if they were previously treated with TZD, treated with more than one diabetic agent or insulin, had hypertension, were pregnant, had active cardiac disease or other major illnesses. Briefly, after a washout period of 6-wk for those participants that were on an anti-diabetic treatment, subjects were treated with rosiglitazone (4 mg twice daily) for a period of 4 months [25]. To minimize potential side effects, medication was initiated below target dose and titrated up over the initial 2 weeks. Blood was collected after 10–12-h overnight fast at baseline and 4 months post treatment. Clinical data of subjects is summarized in Table 1 and all metabolic parameters for subjects were determined using standard techniques as previously described [25,26].

**Table 1.** Baseline characteristics of subjects

N = 7(males)	
BMI (kg/m^2^)	32.9 ± 5.9
Weight (kg)	97.5 ± 17
Fasting glucose (mmol/l)	9.3 ± 2
Fasting insulin (pmol/l)	95.1 ± 38.1
FFA (mmol/l)	0.482 ± 0.102
HbA1c (%)	8.0 ± 1.1
TG (mg/dl)	142 ± 6.7

### 2.3. Metabolic parameters

Glucose tolerance tests (GTT) were performed on mice fasted for 6 h and Insulin tolerance tests (ITT) were performed on non-fasted mice [11,27]. Mice were injected i.p. with glucose (2 g/kg BW) or human insulin (0.75 U/kg, Humulin; Eli Lilly) and blood samples drawn via the tail vein at baseline and 15, 30, 60, 90, and 120 min post-injection. Plasma insulin levels were measured with an insulin assay kit (Mercodia Ultrasensitive Insulin ELISA; Alpco Diagnostics), glucose was monitored with a Glucometer Elite Blood Glucose Metre (Bayer). Plasma FFA was measured with an NEFA-C kit and triglycerides with a Triglyceride E test (Wako).

### 2.4. Sphingolipid analysis

Plasma S1P levels in human subjects was analysed by HPLC-tandem mass spectroscopy as described [11,27] at the Lipidomics core facility, Medical University of South Carolina. Briefly, plasma was spiked with internal standards and extracted into a one-phase neutral organic solvent system (ethyl acetate/isopropanol/water; 60/30/10 vol/vol). The solvents were evaporated, and the sample was reconstituted in methanol and analysed with an HP1100/TSQ 7000 LC/MS system. Quantitative analysis of analytes was performed in positive multiple reaction monitoring mode, based on calibration curves generated by spiking an artificial matrix with known amounts of target analytes, synthetic standards, and internal standards. Plasma S1P in mice were measured using an S1P elisa kit (MyBioSource) according to manufacturers instructions.

### 2.5. Adipose fractionation, flow cytometry and CD11b+ seelction

Adipose fractionation was as previously described [28]. Briefly, epididymal AT (EAT) was washed and minced in PBS containing 0.5% BSA and incubated at 37°C with collagenase (1 mg/ml in PBS/0.5% BSA) for 20 min on a shaking platform. The mixture was filtered through a nylon filter (pore size 250 µm), centrifuged (5 min, t 200 g at 4°C), and floating cells and pellet recovered as mature adipocytes and the SVF, respectively. Flow cytometry of EAT-derived SVF cells was performed as previously described [29,30]. The SVF was incubated in RBC lysis buffer (5 min), resuspended in FACS buffer (PBS containing 1% FCS and 1 mM EDTA) and stained at 4°C for 30 min with fluorophore-labelled monoclonal antibodies to CD11b, CD11c, CD3, CD4 and CD8 (eBioscience) in the presence of Fc receptor blocking antibodies (anti-CD16/CD32; eBioscience). Propidium iodide (Invitrogen) was added for live cell gating. Cells were washed, fixed in 1% formaldehyde, and analysed on an LSR-II cytometer (BD Biosciences) with data processing using FlowJo (Tree Star). CD11b+ cells were positively selected from EAT SVF cells using anti-CD11b paramagnetic microbeads (Miltenyi Biotec).

### 2.6. Real-time quantitative RT-PCR

cDNAs synthesized from total RNA (Ultraspec RNA isolation system; Biotecx Laboratories) were analysed with gene-specific primer sets (Invitrogen) and SYBR Green PCR Master mix (PerkinElmer) in an iCycler (Bio-Rad) [28,29]. Relative gene expression levels were calculated after normalization to β-actin using the ΔΔCT method (Bio-Rad). Separate control experiments demonstrated that the efficiencies of target and reference (i.e. β-actin) amplifications were equal thus validating the use of ΔΔCT for all of the transcripts that were measured.

### 2.7. Histology and immunofluresence

Formalin-fixed, paraffin-embedded sections (6 µm) of adipose and liver were stained with H & E. For immunofluresence, sections of AT were incubated 30 minutes at room temperature with a blocking solution (1X PBS, 10% goat serum, 0.1% Tween-20) with gentle agitation and incubated overnight at 4^ο^C with the primary antibody, anti-mouse S1PR3 at 1:50 dilution (rabbit polyclonal, Santa Cruz Biotechnology, Santa Cruz, CA, USA). Negative controls were done without the primary antibody. Slides were washed 2 × 1 minutes with blocking solution at room temperature with gentle agitation after which the slides were incubated with secondary fluorescent antibody (Invitrogen, Carlsbad, CA, USA) at room temperature for two hours to visualize labelling. Slides were rinsed and mounted with ProLong Gold antifade reagent (Invitrogen, Carlsbad, CA, USA) and analysed using a laser-scaning confocal microscope (Olympus, Tokyo, Japan).

### 2.8. 3T3-L1 adipocyte and oil red O staining

3T3- L1 pre-adipocytes (ATTC) were grown and differentiated as previously described [29]. Briefly, 3T3-L1 pre-adipocytes were grown to confluence in DMEM (Gibco) supplemented with 10% FBS and 1% penicillin/streptomycin. Following 2 days post confluence and clonal expansion adipocyte differentiation was initiated by treating cells for 3 days with 5 µg/mL insulin and 1 µM dexamethasone alone or insulin + dexamethasone + 1 µM sphingosine 1-phosphate (Sigma). Thereafter, media was changed every 3 days with only 100 ng/ml insulin. In some experiments adipocyte differentiation was carried out in the presence or absence of the S1PR1/3 antogonist VPC23019 (1 µM; Sigma). Ten days post-differentiation the cells were fixed with 10% Zn formalin for 20 minutes and permeabilized with 100% propylene glycol for 3 minutes. Fixed cells were then stained with Oil Red O in 100% propylene glycol for 1 hour at 37°C and destained with 60% propylene glycol using gentle agitation for 1 minute. The Oil Red O extractions were done using two 500 µL aliquots of isopropanol combined and absorbance was measured spectrophotometrically at 510 nm.

### 2.9. Statistical analysis

Prism 9.2 software (GraphPad Software, San Diego CA) was used for statistical analysis. Data are presented as mean ± SEM. When two groups were compared (e.g. S1PR3 mRNA between LFD vs. HFD; gene expression between HFD-WT and HFD-S1PR3-/- mice, etc.), data were analysed for statistical significance using a two-tailed unpaired Student’s t test. Repeated measure based parameters (e.g. weight gain over time, GTT, ITT) were analysed using two-way ANOVA for repeated measures followed by the Bonferroni test. Paired Student’s t test was used when comparing S1P levels at baseline and rosiglitazone-treated samples from diabetic human subjects. Correlations between plasma S1P and various glucose homoeostasis parameters in human diabetic subjects were calculated using the Pearson correlation. Statistical significance was defined as p < 0.05. In the figures, statistical significances are denoted with asterisks as follows * =  p < 0.05, ** =  p < 0.01, *** =  p < 0.001.

## 3. Results

### 3.1. Rosiglitazone increases plasma S1P levels in T2D subjects and this increase correlates with its anti-diabetic response

**Figure 1.**
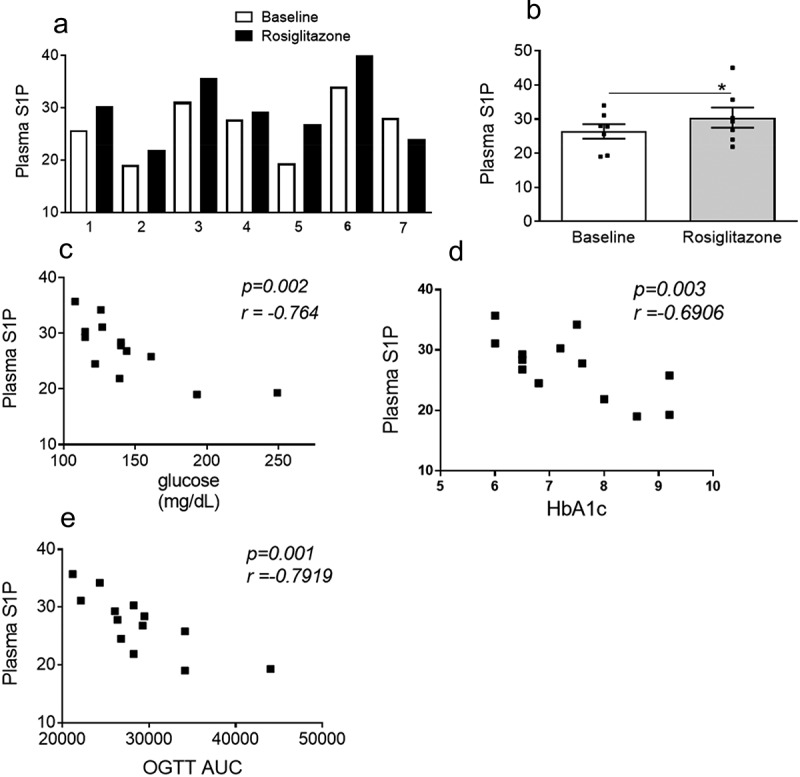
Plasma S1P increases in response to rosiglitazone and correlates with anti-diabetic responses. (a, b) Plasma S1P levels (pmole/100 µL) at baseline and following treatment with rosiglitazone. For a and b, n = 7 ± SEM, *P < 0.05, baseline versus rosiglitazone. (c,d,e) Baseline and post-rosiglitazone treated S1P values were used to determine correlations of plasma S1P with metabolic parameters of body weight, fasting plasma glucose, plasma HbA1c and OGTT AUC respectively. For c-e, n = 12–14.

Thiazolidinediones (TZDs) that act through the nuclear receptor peroxisome proliferator-activated receptor-ᵞ (PPARᵞ) are potent insulin sensitizers and highly effective oral medications for T2D. To determine if the insulin sensitizing effects of TZDs are associated with changes in S1P levels in vivo, we measured plasma S1P in a small cohort of obese T2D subjects at baseline and after 4 months of rosiglitazone treatment. Baseline metabolic characteristics of subjects prior to treatment is indicated in Table 1. A significant increase in plasma S1P was observed post-rosiglitazone treatment compared with baseline levels (Figure 1(a, b)). Of the seven patients that were studied, six of them showed an increase while one showed a modest decrease in plasma S1P. To our knowledge, this is the first in vivo demonstration that rosiglitazone induces plasma S1P in human subjects which suggests that it may contribute to the anti-diabetic outcomes of this drug. In this cohort of patients, we also determined the extent to which S1P levels correlated with anti-diabetic outcomes. We found that high S1P correlated with low glucose, HbA1C and OGTT/AUC (Figure 1(c–e)). These data suggest a potential mechanistic role for S1P in mediating protective metabolic outcomes relating to glucose homeostasis and insulin-sensitivity.

### 3.2. S1PR3 expression is increased in adipose tissues (AT) of high fat diet (HFD)-induced obese mice

**Figure 2.**
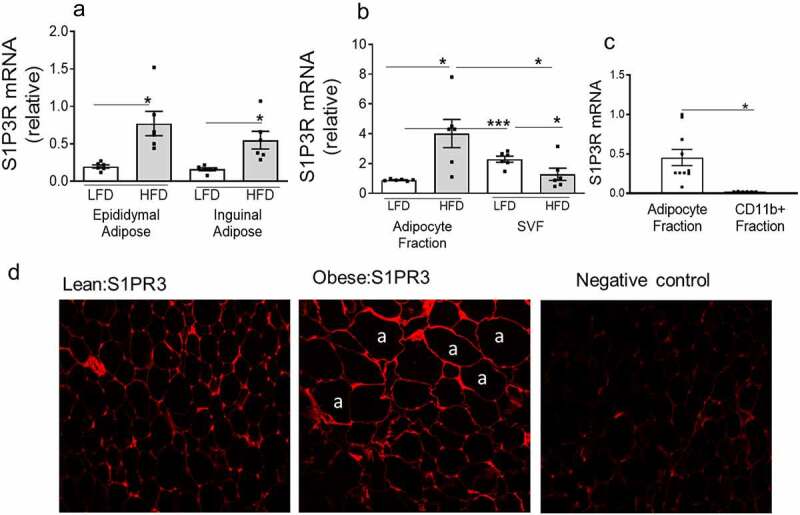
S1PR3 expression is increased in adipose tissues of high fat diet (HFD)-induced obese mice. (a) S1PR3 mRNA expression in EAT and SAT (Inguinal) of male C57BL/6 J mice on either a low fat diet (LFD, 10% fat) or a high fat diet (HFD, 60% fat) for 16 weeks. (b) S1PR3 mRNA in isolated adipocytes and SVF fraction from EAT of LFD and HFD fed mice. For (a) and (b) n = 6 ± SEM. *P < 0.05, ***P < 0 .001, LFD versus HFD. (c) S1PR3 mRNA in adipocytes and in AT macrophages from EAT of HFD fed mice. For (c) n = 3 ± SEM. *P < 0 .05, adipocytes versus CD11+ fraction cells.(d) Representative immunofluorescence images from EAT of lean (LFD) and obese (HFD) mice. Showing staining for S1PR3 around the periphery of adipocyte (‘a’) cell membranes.

To obtain mechanistic insights into the role of S1P in IR and T2D we used a model of HFD-induced obesity in C57BL/6 J mice that mimics the metabolic syndrome, obesity and impaired glucose homeostasis that occurs in humans. When WT male C57BL/6 J mice are fed a HFD they gain weight, become glucose intolerant and insulin resistant, and develop an elevation of fasting glucose levels accompanied by a moderate to distinct increase in fasting plasma insulin levels (Supplementary Figure S1a-e). HFD-fed C57BL/6 J mice also exhibit local adipose inflammation and ectopic lipid deposition in the liver causing hepatic steatosis (Supplementary figure S1f-e). We measured S1PR3 gene expression in AT of HFD-induced obese male C57BL/6 J mice. Relative to mice that were fed a low-fat diet (LFD), S1PR3 mRNA levels were significantly higher in both the EAT and the inguinal subcutaneous AT (SAT; Figure 2(a)). Adipocytes from the EAT were isolated using collagenase digestion and separated from the rest of the stromal vascular fraction (SVF) cells and S1PR3 gene expression determined in these cell fractions. While S1PR3 expression was higher in adipocytes of HFD-fed mice its expression was decreased in the SVF cells of HFD-fed mice compared to its lean counterpart (Figure 2(b)). The SVF cells contain multiple cell types including pre-adipocytes, endothelial cells, smooth muscle cells, mesenchymal stem cells, macrophages, and other immune cells. We also compared the cellular expression of S1PR3 in adipocytes versus macrophages in EAT of HFD-fed mice. Again, S1PR3 gene expression was relatively higher in adipocytes compared with ATM (Figure 2(c)). We performed immunofluorescence studies for S1PR3 expression in EAT sections from lean and obese mice. In the obese mice, more intense S1PR3 staining in adipocytes were clearly observed around the periphery of adipocyte cell membranes (Figure 2(d)).

### 3.3 Adiposity and glucose *homeostasis* are impaired in mice deficient in S1PR3

**Figure 3.**
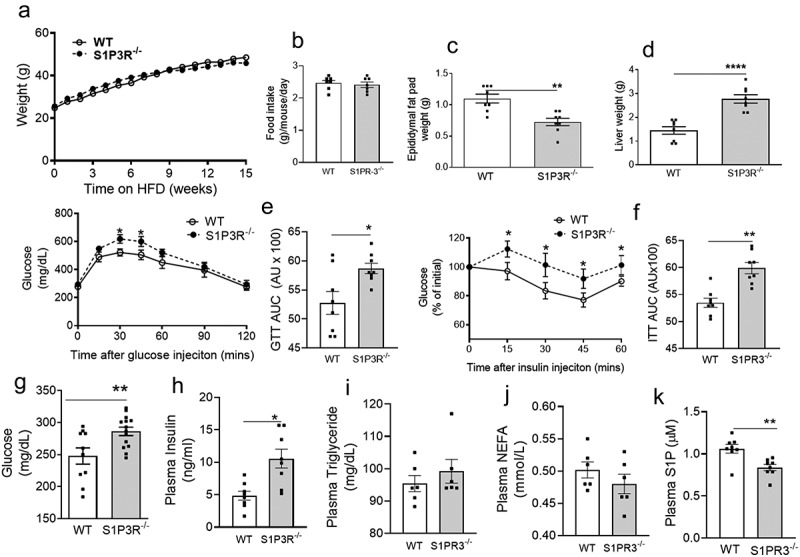
Adiposity and glucose homoeostasis are impaired in mice deficient in S1P3R. (a) Body weights and food intake (b) of male S1PR3^−/−^ mice and WT littermates on a HFD over 15 weeks n = 16 ± SEM. (c) EAT weights of S1PR3^−/−^ mice and WT littermates after 15 weeks on the HFD. (d) Liver weights of S1PR3^−/−^ mice and WT littermates after 15 weeks on the HFD. For c and d, n = 16 ± SEM. **P < 0.01, ****P < 0 .0001, WT versus S1PR3^−/−^ mice. (e, f) Glucose tolerance test/GTT-AUC and insulin tolerance tests/ITT AUC respectively of HFD-fed WT and S1PR3^−/−^ mice respectively. n = 8 ± SEM. *P < 0 .05, **P < 0.01, WT versus S1PR3^−/−^ mice. (g,h,i,j,k) Fasting blood glucose, plasma insulin, plasma triglyceride, plasma NEFA and plasma S1P respectively of HFD-fed WT and S1PR3^−/−^ mice. n = 6–8 ± SEM. *P < 0 .05, **P < 0.01, WT versus S1PR3^−/−^ mice.

To directly assess the contribution of S1PR3 to obesity and its metabolic consequences, we compared the physiological and biochemical consequences of a 16-wk HFD on WT and S1PR3^−/−^ mice. HFD-fed WT and S1PR3^−/−^ mice gained weight at a similar rate (Figure 3(a)), but showed no difference in food intake (Figure 3(b)). However, the S1PR3^−/−^ mice had lower EAT weights and higher liver weights (Figure 3(c, d)), indicating a blunted lipid storing ability of the AT and shunting of lipids ectopically to the liver resulting in a phenotype of partial lipodystrophy. We next determined the extent to which glucose homeostasis was regulated in HFD-fed WT and S1PR3^−/−^ mice. In glucose tolerance tests (GTT), HFD-fed S1PR3^−/−^ mice were less efficient in clearing an intraperitoneal bolus of glucose compared to HFD-WT littermate mice, and the AUC was also higher in the HFD-fed S1PR3^−/−^ mice (Figure 3(e)). Compared to HFD-WT mice, HFD-fed S1PR3^−/−^ mice also demonstrated decreased insulin sensitivity as shown by the greater resistance to insulin-mediated suppression of plasma glucose in insulin tolerance tests (ITT) and increased AUC (figure 3(f)). Glucose intolerance and IR observed in HFD-fed S1PR3^−/−^ mice were accompanied by an increase in fasting levels of plasma glucose and insulin (Figure 3(g, h)). However, plasma triglycerides and FFA were similar in HFD-WT and S1PR3^−/−^ mice (Figure 3(i, j)). We next determined if these metabolic abnormalities of the HFD-fed S1PR3^−/−^ were accompanied by changes in plasma S1P levels.

Plasma S1P was significantly decreased in HFD-fed S1PR3^−/−^ mice compared to its WT counterparts (Figure 3(k)).

### 3.4. Loss of S1PR3 increases adipose tissue inflammation and immune cell accumulation

**Figure 4.**
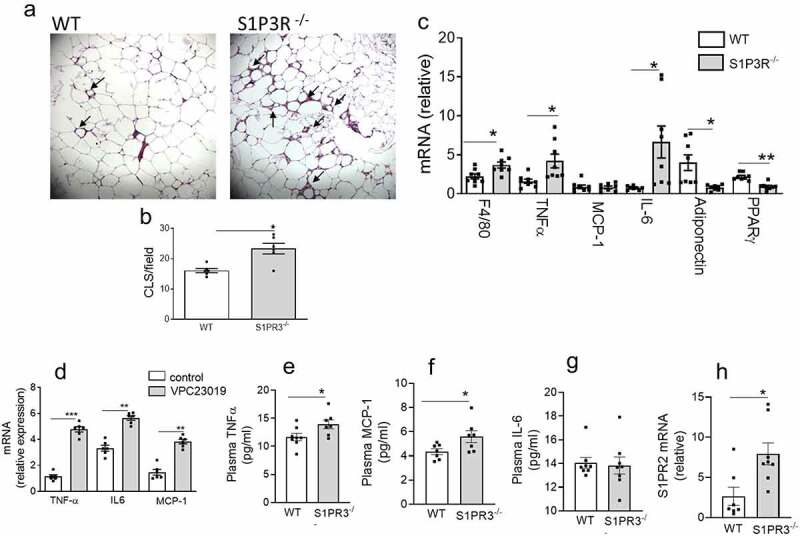
Loss of S1PR3 increases adipose tissue inflammation. (a) Representative paraffin-embedded H&E-stained sections of EAT from HFD-fed WT and S1PR3^−/−^ mice. Arrows point to ‘crown-like structures’. (b) Quantification of CLS/microscopic field of H & E stained EAT sections from HFD-fed WT and S1PR3^−/−^ mice. n = 6 ± SEM. *P < 0 .05, WT versus S1PR3^−/−^ mice. (c) Cytokine/chemokine/adipokine expression in EAT of HFD-fed WT and S1PR3^−/−^ mice. n = 8 ± SEM. *P < 0.05, **P < 0.01, WT versus S1PR3^−/−^ mice. (d) Cytokine/chemokine gene expression in 3T3-L1 adipocytes in response to the S1PR1/S1PR3 antagonist VPC23019. n = 6 ± SEM. **P < 0 .01, ***P < 0 .001, control versus VPC23019. (e,f,g,h) Plasma TNF-α, plasma MCP-1, plasma IL-6 and EAT S1PR2 mRNA respectively of HFD-fed WT and S1PR3^−/−^ mice. n = 6–8 ± SEM. *P < 0 .05 WT versus S1PR3^−/−^ mice.

Obesity is associated with chronic inflammation of the AT with increased expression of pro-inflammatory cytokines/chemokines and the accumulation of macrophages and other immune cells including specific T-cell subsets [3–5]. These changes cause adipose dysfunction and are mechanistically related to local and systemic IR/T2D [3–5]. We determined if the impaired glucose homeostasis observed in HFD-fed S1PR3^−/−^ mice was mechanistically related to an adipose inflammatory response. Hallmarks of proinflammatory processes in AT can be histologically identified in crown-like structures (CLS), which are foci of dying adipocytes surrounded by macrophages and other immune cells. H&E staining indicate that CLS were higher in the EAT of HFD-fed S1PR3^−/−^ mice compared with HFD-fed WT mice (Figure 4(a,b)). Gene expression analysis identified transcriptional changes in the EAT of HFD-fed S1PR3^−/−^ mice that were consistent with an inflammatory phenotype (Figure 4(c)). Specifically, the macrophage marker F4/80 was increased as well as TNF-α and IL-6 (Figure 4(c)). We also observed a decrease in the insulin sensitizing protein, adiponectin, and a significant decrease in PPARγ, a key adipogenic transcription factor, in the EAT of HFD-fed S1PR3^−/−^ mice (Figure 4(c)). In confirmation of the in vivo phenotype, treatment of 3T3-L1 adipocytes with the S1PR1/S1PR3 antagonist VPC23019 also resulted in increased expression of TNF-α, IL-6 as well as MCP-1 (Figure 4(d)). Inflammatory cytokines in the plasma of HFD-S1PR3^−/−^ and HFD-WT mice showed that TNF-α and MCP-1 levels were elevated in HFD-S1PR3^−/−^ mice, while no changes were observed for IL-6 (Fig ure 4(e,f,g). Since S1PR2 signalling has been shown to exacerbate metabolic/inflammatory dysfunction [45], we also determined S1PR2 gene expression in epididymal AT of S1PR3^−/−^ mice, and found that S1PR2 mRNA levels were significantly elevated in the epididymal AT of HFD-fed S1PR3^−/−^ mice.(Figure 4(h)).

**Figure 5.**
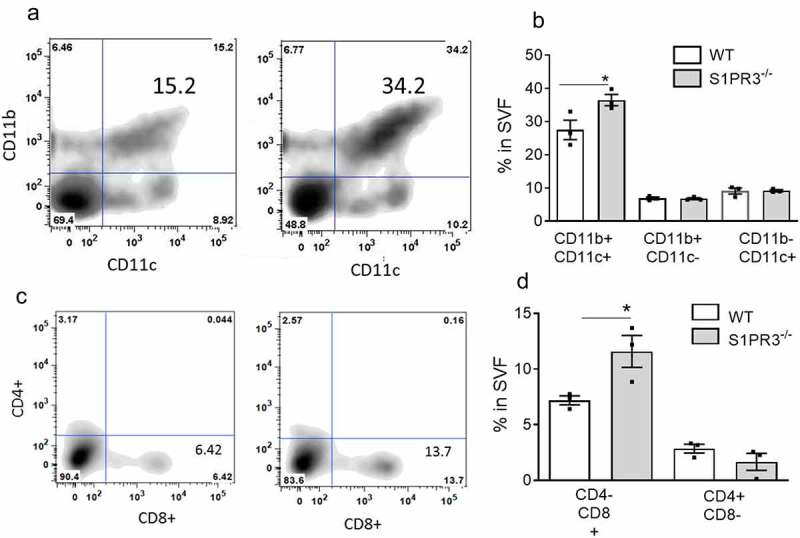
Loss of S1PR3 increases immune cell accumulation in adipose tissue. (a,b) FACS quantification of CD11b+/CD11 c+ and CD11b+/CD11 c- macrophage populations in EAT SVF cells from HFD-fed WT and S1PR3^−/−^ mice. (c,d) FACS quantification of CD4-/CD8+ and CD4+/CD8- T cell populations in EAT SVF cells from HFD-fed WT and S1PR3^−/−^ mice. For FACS quantification of macrophage and T cell populations 2–3 mice were pooled. n = 3 ± SEM. *P < 0 .05 for WT and S1PR3^−/−^ mice.

AT macrophage populations in obesity are extremely heterogeneous and plastic, and an increase in both pro-inflammatory M1-like and anti-inflammatory M2-like macrophages as well as populations with mixed M1/M2-like surface marker profiles have been described [31–34]. By FACS analysis we found that a population of macrophages with the surface marker signature CD11b+/CD11c+ (M1-like macrophages) were significantly increased in the EAT of HFD-fed S1PR3^−/−^ mice compared to HFD-fed WT mice (Figure 5(a,b)) while no changes were observed in the macrophage population expressing CD11b+/CD11c- (M2-like macrophages). A markedly higher number of CD8+ T effector cells was observed in the EAT of HFD-fed S1PR3^−/−^ compared with HFD-fed WT mice whereas no changes were observed in the number of CD4+ cells (Figure 5(c,d)).

### 3.5. Loss of S1PR3 causes hepatic steatosis and inflammation

**Figure 6.**
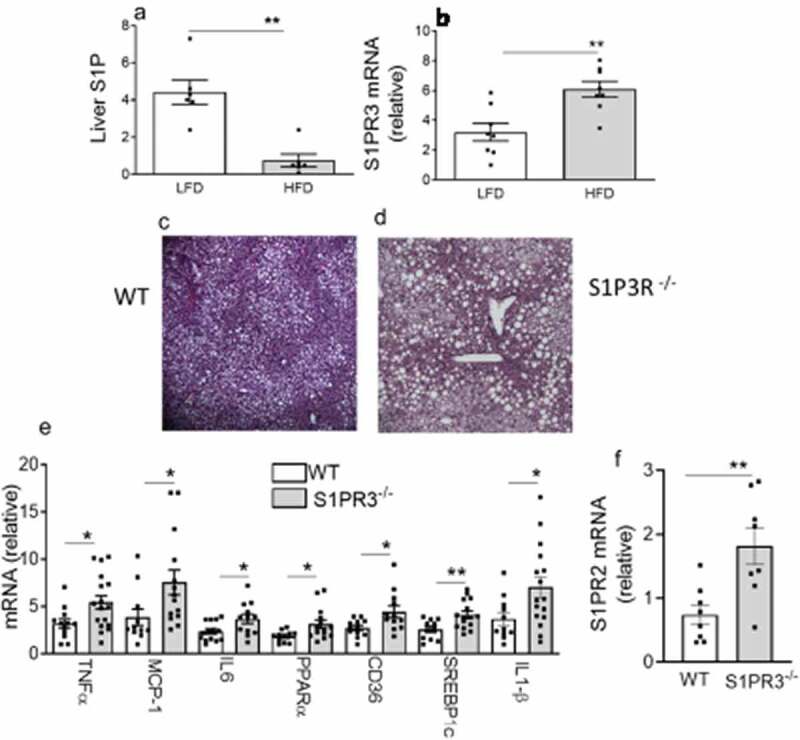
Loss of S1P3R causes hepatic steatosis and inflammation. (a) Liver S1P content in male C57BL/6 J mice fed a LFD or HFD for 16 weeks. n = 6 ± SEM. **P < 0 .01 for LFD versus HFD. (b) S1PR3 gene expression in male C57BL/6 J mice after 16 weeks on a LFD or HFD. n = 6 ± SEM. **P < 0 .01 for LFD versus HFD. (c,d) Representative paraffin-embedded H&E-stained sections of liver from HFD-fed WT and S1PR3^−/−^ mice. (e) Gene expression of cytokines, chemokines and genes related to fatty acid transport/storage in the livers of HFD-fed WT and S1PR3^−/−^ mice. n = 8 ± SEM. *P < 0.05, **P < 0.01, WT versus S1PR3^−/−^ mice. (f) Liver S1PR2 mRNA of HFD-fed WT and S1PR3^−/−^ mice. n = 8 ± SEM. **P < 0 .01 for WT versus S1PR3^−/−^ mice.

Obesity also causes increased inflammation in the liver and hepatic steatosis that contributes to IR and impaired glucose tolerance. We initially measured S1P levels in the livers of mice fed either an HFD or LFD and found a significant decrease in S1P in HFD-fed mice, suggesting a protective effect of S1P in the liver (Figure 6(a)). Moreover, the decrease in the weight of EAT observed in HFD-fed S1PR3^−/−^ mice accompanied a significant increase in the weight of the liver suggesting the involvement of the liver in the impaired glucose homoeostasis of the HFD-fed S1PR3^−/−^ mice. We therefore next determined how the expression of S1PR3 is regulated in the livers of HFD-induced obese mice and its role in hepatic steatosis and inflammatory pathways. S1PR3 gene expression was significantly elevated in mice fed a HFD when compared to mice on a LFD (Figure 6(b)). In HFD-fed S1PR3^−/−^ mice, H&E stained sections of the liver indicated elevated lipid deposition when compared with HFD-fed WT mice (Figure 6(c,d)). Mechanistically, increased hepatic steatosis in HFD-fed S1PR3^−/−^ mice is likely associated with the observed high expression of the fatty acid transporter gene, CD36, and the lipogenic transcription factor, sterol regulatory element binding protein 1 c (SREBP1c), with additional involvement of inflammatory components including TNF-α, MCP-1, IL-6 and IL-1β (Figure 6(e)). The dysfunctional changes in the liver of HFD-fed S1PR3^−/−^ mice was also accompanied by increased expression of S1PR2 (Figure 6(f)).

### 3.6. S1P regulates adipogenesis

**Figure 7.**
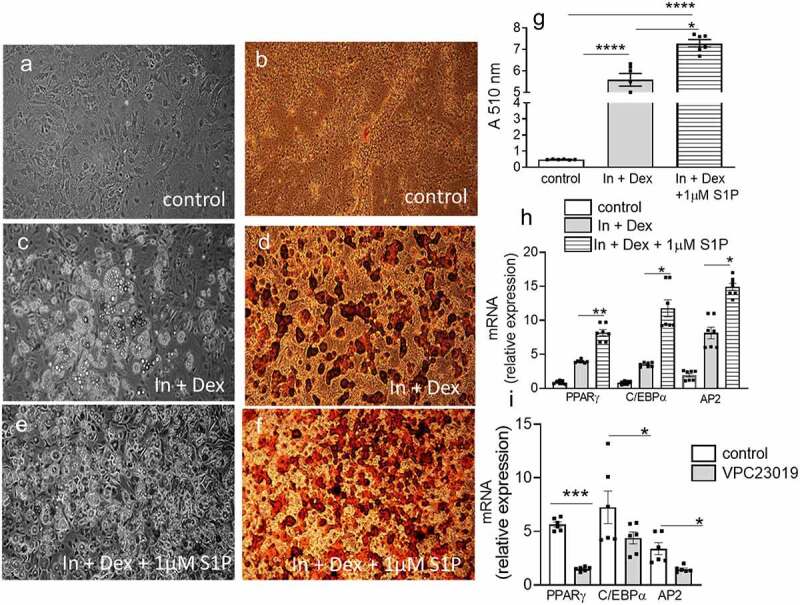
S1P regulates adipogenesis. (a,c,e) Representative phase-contrast images of 3T3-L1 adipocytes (control, Insulin + dexamethasone, Insulin + dexamethasone + 1 μM S1P) 10 days post differentiation. (b,d,f) Representative Oil-Red O stained 3T3-L1 adipocytes (control, Insulin + dexamethasone, Insulin + dexamethasone + 1 μM S1P) 10 days post differentiation. (g) Spectrophotometric absorbance readings of isopropanol extracted Oil-Red O stained adipocytes under the above conditions. n = 6 ± SEM. ****P < 0 .0001, control versus treatment as indicated. (h) adipogenic gene expression in differentiating 3T3-L1 adipocytes under the above conditions. n = 6 ± SEM. *P < 0.05, **P < 0.01, Insulin + dexamethasone versus Insulin + dexamethasone + 1 μM S1P. (i) Adipogenic gene expression in differentiating 3T3-L1 adipocytes in response to the S1PR1/S1PR3 antogonist VPC23019. n = 6 ± SEM. *P < 0.05, ***P < 0.001, control versus VPC23019.

Thiazolidinediones such as rosiglitazone are PPARγ activators that increase insulin sensitivity and adipogenesis. Improved IR of rosiglitazone-treated T2D subjects was associated with increased S1P, suggesting the possibility that S1P signalling maybe mechanistically linked with improved IR and adipogenesis. We directly determined if S1P regulates adipogenesis using the 3T3-L1 adipocyte model. Confluent 3T3-L1 preadipocytes were either treated with regular media, induced to differentiate into adipocytes in the presence of an inducing cocktail of Insulin + dexamethasone or in the presence of the inducing cocktail together with S1P. Ten days after induction of differentiation, no apparent adipocytes were observed in cells treated with the control media (Figure 7(a,b)), whereas adipogenesis was readily seen by both phase contrast and Oil Red O staining in cells treated with Insulin + dexamethasone (Figure 7(c,d)). When S1P was included in the differentiation cocktail, adipogenesis was dramatically and significantly increased over and above that observed with Insulin + dexamethasone alone (Figure 7(e,f)). Alcohol extraction of Oil Red O from adipocytes and subsequent quantification additionally show the significant enhancement of adipogenesis in the presence of S1P (Figure 7(g)). In these experiments, early adipogenesis transcription factors C/EBPα and PPARγ as well as the PPARγ target gene AP2 were increased in the presence of S1P compared to Insulin + dexamethasone only (Figure 7(h)). The decreased EAT weight and decreased adipose PPARγ expression in HFD-fed S1PR3^−/−^ mice may indicate that S1P promotes adipogenesis via S1PR3 signalling. We determined whether blocking S1PR3 signalling might interfere with the adipogenic gene expression program. Treatment of 3T3-L1 pre-adipocytes with VPC23019 (an antagonist of S1PR3/1) during adipogenesis resulted in decreased expression of C/EBPα, PPARγ and AP2 (Figure 7(i)).

## 4. Discussion

While ceramide has been confirmed to be involved in the pathogenesis of obesity and associated disease sequelae (i.e. IR/T2D/hepatic steatosis) [11], the function of S1P is neither extensively investigated nor well defined and its role seems to be controversial depending on the cell type and expression pattern of its receptors. The major results of the present study were; 1) In T2D subjects plasma S1P was significantly increased in response to the anti-diabetic drug, rosiglitazone; 2) Plasma S1P correlated with measures of improved glucose homoeostasis; 3) Glucose homeostasis was exacerbated in HFD-fed S1PR3^−/−^ mice compared to HFD-fed WT counterparts; 4) The worsened metabolic phenotype of HFD-fed S1PR3^−/−^ mice was mechanistically related to inflammation in the adipose and liver and increased hepatic steatosis; 5) S1P promoted adipogenesis in 3T3-L1 preadipocytes, potentially via S1P3. This study therefore demonstrates that S1P is protective in the setting of obesity and T2D, and, that its protective effects are partly due to signalling via the S1PR3 in AT and liver.

Some studies have reported that plasma S1P levels are increased in murine and human obesity [35,36]. However, it is unclear if S1P is causative to the pathogenesis of IR and T2D and/or if S1P is protective and exhibits a compensatory increase under these conditions. We demonstrate that plasma S1P levels are elevated in response to the anti-diabetic drug, rosiglitazone, and correlates with improved glucose homoeostasis suggesting a protective role for S1P in metabolic homeostasis. Indeed, some clues in the literature likewise indicate a protective function for S1P. S1P is inversely associated with the progression of T2D [14], Plasma S1P in HDL are lower in T2D compared with controls, and HDL-associated S1P was inversely correlated with HbA1c levels [37]. S1P levels in HDL is an independent protector against the development of T2D associated cardiovascular disease [37–39]. While the underlying mechanisms of these protective effects are not well defined, the well-known bioactive properties of S1P including its pro-survival, anti-apoptotic, anti-inflammatory and immune cell trafficking properties are likely to play a role. S1P also regulates the maturation and function of pancreatic-β cells and promotes glucose-stimulated insulin secretion [40]. The insulin sensitizer, adiponectin, diminished ceramide accumulation and stimulated S1P, which enhanced the positive metabolic effects of adiponectin [18,41]

Whereas weight gain was similar in HFD-fed S1PR3^−/−^ mice and HFD-fed WT mice, body fat distribution and glucose homeostasis differed significantly between these mice. HFD-fed S1PR3^−/−^ showed a fat distribution that resembled a phenotype of partial lipodystrophy with redistribution of lipids from AT to the liver resulting in decreased EAT weight with increased liver weight and steatosis. These changes were associated with exacerbated IR and glucose intolerance in HFD-fed S1PR3^−/−^ mice. The decreased adipose weights and intensified metabolic phenotype of the HFD-fed S1PR3^−/−^ mice, is consistent with studies showing that adipose dysfunction and not adiposity per-se contributes to metabolic abnormalities [42] [43,44]. Previously, loss of S1PR2 signalling decreased weights of epididymal fat pads but improved glucose tolerance in HFD-fed mice [45]. Here, however, the extent to which the decrease in AT weight in HFD-fed S1PR2^−/−^ mice impacted liver pathology was not indicated [45]. Interestingly, we found that S1PR2 mRNA levels were significantly elevated in both the liver and adipose of HFD-fed S1PR3^−/−^ mice. Therefore, it is likely that the increase in insulin resistance and glucose intolerance observed in S1PR3^−/−^ mice maybe due to a combined dual effect of the loss of S1PR3 signalling and increased expression of S1PR2.

Macrophages are the predominant immune cells that accumulate in AT of obese mice and humans, and, obese AT shows high expression of both M2-like and proinflammatory M1-like macrophages [31–34] and areas of ‘crown-like structures’ (CLS) contain many macrophages, most of which are M1-like [46,47]. While a number of cues that promote macrophage trafficking to AT have been identified, mechanisms preventing excessive accumulation of macrophages are relatively unknown. Increased CLS were observed in EAT of HFD-fed S1PR3^−/−^ mice and M1-like AT macrophage populations were also higher in HFD-fed S1PR3^−/−^ mice compared with HFD-fed WT mice. While macrophages are an important source of AT inflammation, changes in adipose T cell populations also regulate obesity-associated inflammation and insulin sensitivity. Multiple studies report increased numbers of CD8+ effector T cells in the AT of obese rodents and humans and depletion of CD8+ T cells alleviates AT inflammation and insulin resistance in obese mice [48–50]. In keeping with these observations, we found increased levels of CD8+ T cells in EAT of HFD-fed S1PR3^−/−^ mice with no changes in CD4+ T cell populations. Our results suggest that S1P-S1PR3 signalling inhibits migration of macrophages and T-cells to AT and thus identifies a novel negative regulator of excessive adipose immune cell accumulation. In parallel with increased macrophages and T-cells, the pro-inflammatory cytokines, TNF-α and IL-6 were also elevated in EAT of HFD-fed S1PR3^−/−^ mice, whereas the insulin sensitizers adiponectin and PPAR_ϒ_ were decreased. Anti-inflammatory functions have been reported for S1P-S1PR3 signalling in other biological systems. S1P lyase inhibition increases S1P, reduces cytokine production including TNF-α and IL-6 and protects against sepsis via the S1P-S1PR3 axis [51]. In sepsis models, S1P3^−/−^ mice had reduced survival rates concomitant with increased inflammatory response of TNF-α and IL-6 [52]. The phosphorylated fingolimod pFTY720 depends on S1PR3 to protect astrocytes against oxygen–glucose deprivation-induced neuroinflammation, due to inhibiting a TLR2/4‐PI3K‐NFκB signalling pathway [53]. S1PR3 in the medial prefrontal cortex promotes stress resilience by reducing inflammatory cytokine TNF-α production [54]. FTY720 exerts a general anti-inflammatory potential by reducing immune cell adhesion to endothelial cells through activation of S1PR3 [55]. Moreover, S1PR3 signalling was also shown to regulate the recruitment of anti-inflammatory regenerative monocytes that can potentially enhance healing outcomes [56].

HFDs in WT C57BL/6 J mice causes hepatic steatoses and inflammation, and some studies suggest the contribution of sphingolipids such as ceramide in this process. However, manipulating ceramide synthesis/degradation will also invariably result in changes in S1P which may also be involved in the pathogenesis of hepatic steatosis and inflammation. We observed that hepatic steatosis in HFD-fed WT C57BL/6 J mice was associated with a dramatic decrease in S1P, which may suggest that S1P protects from hepatic steatoses. In this respect, the insulin sensitizing hormone, adiponectin, lowers ceramide levels, increases S1P in the liver and improves insulin-signalling [18]. However, the decrease in S1P in HFD-fed WT C57BL/6 J mice may also be secondary to liver’s impairment.

Steatosis was dramatically increased in HDF-fed S1PR3^−/−^ mice when compared with HFD-WT counterparts. Transcriptional activation of inflammatory cytokines (TNF-α, IL-6, IL-1β) and macrophage markers/chemokines (F4/80, MCP-1) known to cause liver injury and inflammation were also dramatically increased in the livers of HFD-fed S1PR3^−/−^ mice. The exacerbated hepatic steatosis and inflammation observed in HFD-fed S1PR3^−/−^ mice above what was observed for HFD-fed WT mice suggests that S1P-S1PR3 signalling confers protection from lipid accumulation and inflammation in the liver. In our study, increased hepatic steatosis in HFD-fed S1PR3^−/−^ mice was mechanistically related to elevated expression of CD36, a fatty acid transporter and the lipogenic transcription factor SREBP1c. In previous studies, FTY720 decreased murine non-alcoholic hepatic steatosis and inflammation [57]. However, the actual mechanisms of action of FTY720 in these studies are unclear. While FTY720 is an agonist at S1PR1 and S1PR3, it acts as a functional antagonist at S1PR1 due to internalization of the receptor. Such a desensitization and internalization of S1PR3 in response to FTY720 has not been shown, and therefore FTY720 appears to be a true agonist at S1PR3. Our data, however, show a clear function for S1PR3 in hepatic steatosis and inflammation associated with diet-induced obesity.

Previous studies of S1P and its receptors in the regulation of adipogenesis have been inconsistent and controversial with both anti-adipogenic [58,59], and pro-adipogenic functions [60] being described for S1P. Our data are consistent with studies showing a pro-adipogenic role for S1P. It is also consistent with studies demonstrating that S1P is a PPAR_ϒ_ ligand [61,62], and, that S1P increases glucose uptake through transactivation of insulin receptors [63]. No doubt, the complex nature S1P expression and signalling (i.e. intracellular vs. extracellular) and cell-specific expression pattern of its receptors with sometimes opposing functions coupled to multiple downstream signalling pathways are likely to contribute to these different outcomes. Moreover, the experimental protocols are not always standardized and relative expression of S1PRs may vary in the cell lines used in the various laboratories. Given the cell proliferative effects of S1P, it may be important in the clonal proliferation of confluent pre-adipocytes, a pre-requisite for adipogenesis. Therefore, the timing and concentration of S1P addition in adipogenesis experiments are crucial and may lead to divergent outcomes. With respect to the contribution of S1PRs our results for the first time point to a pro-adipogenic role for S1PR3. Here as well, opposing outcomes have been reported for other S1PRs receptors including S1PR1 and S1PR2 [45,59,64,65]. Clearly, more rigorous and standardized experiments are needed to unequivocally clarify the role of S1P/S1PRs in adipogenesis and metabolic function.

Ceramide and S1P are intimately linked metabolic pathways with opposing roles in many biological systems; ceramide is mostly described as a cell death activator while S1P promoted survival. The spatial and temporal expression of the ceramide-S1P rheostat and expression levels of the various S1PRs in metabolic tissues will ultimately govern the function of these signalling molecules and metabolic outcomes of IR, T2D and hepatic steatosis. Our findings presented here indicate an insulin sensitizing/anti-diabetic role for S1P, and, that the S1P-S1PR3 signalling axis is a control point which uniquely maintains healthy adipose function. It does so via its pro-adipogenic effects and is a negative regulator that protects from excessive adipose inflammation during weight gain. In the liver, S1P-S1PR3 signalling defends against hepatic steatosis and inflammation. Consequently, S1P-S1PR3 signalling in the adipose and liver contributes to the maintenance of metabolic homeostasis at multiple key regulatory pathways and may offer potential opportunities for clinical intervention.

## Supplementary Material

Supplemental Material

## Data Availability

The data that support the findings of this study are available from the corresponding author, [FS; SC], upon reasonable request.
