# Supplementary material for: S1P/S1PR3 signalling axis protects against obesity-induced metabolic dysfunction
Source: Adipocyte. 2022 Jan 30;11(1):69–83. doi: 10.1080/21623945.2021.2021700 (PMC8803104; doi:10.1080/21623945.2021.2021700)
Supplement: Supplemental Material [file KADI_A_2021700_SM2160.zip › supplementary/downloadFromZipFile.pdf]

S1P/S1PR3 signaling axis protects against obesity-induced metabolic dysfunction

Sagarika Chakrabarty, Quyen Bui, Leylla Badeanlou, Kelly Hester, Jerold Chun, Wolfram Ruf, Theodore P Ciaraldi, Fahumiya Samad

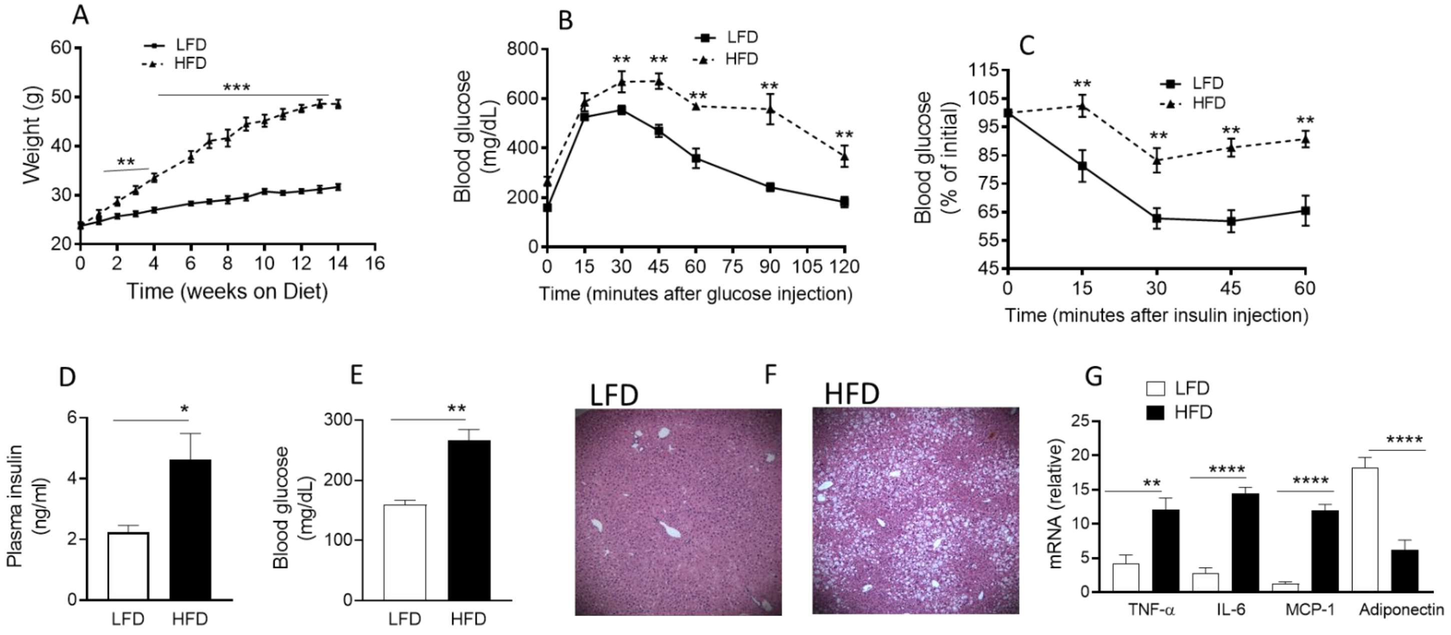

Supplementary Fig. 1: Metabolic phenotyping of HFD-fed C57BL/6J mice. (A-C) Body weights, GTT and ITT respectively of HFD-fed C57BL/6J mice compared to its LFD-fed counterparts. (D-E) fasting plasma insulin and blood glucose in HFD-C57BL/6J mice compared to its LFD-fed . Representative H & E stained sections of livers from LFD and HFD- fed mice. (G) Epididymal Adipose tissues expression of cytokines and adiponectin in LFD-fed and HFD-fed mice. For A-G, n =8 ± SEM. *P < 0.05, **P < 0.01, ***P < 0 .001, ****P < 0 .0001, LFD versus HFD.
